# Resolving Recalcitrant Clades in the Pantropical Ochnaceae: Insights From Comparative Phylogenomics of Plastome and Nuclear Genomic Data Derived From Targeted Sequencing

**DOI:** 10.3389/fpls.2021.638650

**Published:** 2021-02-04

**Authors:** Julio V. Schneider, Juraj Paule, Tanja Jungcurt, Domingos Cardoso, André Márcio Amorim, Thomas Berberich, Georg Zizka

**Affiliations:** ^1^Department of Botany and Molecular Evolution, Senckenberg Research Institute and Natural History Museum Frankfurt, Frankfurt am Main, Germany; ^2^Entomology III, Department of Terrestrial Zoology, Senckenberg Research Institute and Natural History Museum Frankfurt, Frankfurt am Main, Germany; ^3^Institute of Ecology, Evolution and Diversity, Goethe University, Frankfurt am Main, Germany; ^4^Instituto de Biologia, Universidade Federal da Bahia (UFBA), Salvador, Brazil; ^5^Universidade Estadual de Santa Cruz (UESC), Ilhéus, Brazil; ^6^Herbário André Maurício Vieira de Carvalho, CEPEC, CEPLAC, Itabuna, Brazil; ^7^Senckenberg Biodiversity and Climate Research Center, Lab-Center, Frankfurt am Main, Germany

**Keywords:** hybrid enrichment, off-target reads, phylogenomics, phylogenetic conflict, plastome, Malpighiales, taxon sampling

## Abstract

Plastid DNA sequence data have been traditionally widely used in plant phylogenetics because of the high copy number of plastids, their uniparental inheritance, and the blend of coding and non-coding regions with divergent substitution rates that allow the reconstruction of phylogenetic relationships at different taxonomic ranks. In the present study, we evaluate the utility of the plastome for the reconstruction of phylogenetic relationships in the pantropical plant family Ochnaceae (Malpighiales). We used the off-target sequence read fraction of a targeted sequencing study (targeting nuclear loci only) to recover more than 100 kb of the plastid genome from the majority of the more than 200 species of Ochnaceae and all but two genera using *de novo* and reference-based assembly strategies. Most of the recalcitrant nodes in the family’s backbone were resolved by our plastome-based phylogenetic inference, corroborating the most recent classification system of Ochnaceae and findings from a phylogenomic study based on nuclear loci. Nonetheless, the phylogenetic relationships within the major clades of tribe Ochnineae, which comprise about two thirds of the family’s species diversity, received mostly low support. Generally, the phylogenetic resolution was lowest at the infrageneric level. Overall there was little phylogenetic conflict compared to a recent analysis of nuclear loci. Effects of taxon sampling were invoked as the most likely reason for some of the few well-supported discords. Our study demonstrates the utility of the off-target fraction of a target enrichment study for assembling near-complete plastid genomes for a large proportion of samples.

## Introduction

Next-Generation Sequencing (NGS) together with hybridization-based enrichment of selected target loci has emerged as a powerful tool for unraveling phylogenetic relationships of organisms that hitherto remained intractable with traditional Sanger sequencing. Besides the massive amount of obtained sequence data, a major advantage of the NGS short-read technologies (e.g., Illumina) is that it also opened the field to sequencing museum specimens with highly degraded DNA (Staats et al., 2013; Bakker, 2017). This was a major advancement especially for the study of tropical plant groups which often rely on such material.

Instead of sequencing whole genomes, target enrichment seeks to reduce the genomic complexity by selecting only a small portion of the genome, thereby drastically reducing sequencing costs and computational burden (Cronn et al., 2012). During target enrichment, a preselected set of loci is captured and enriched using locus-specific RNA probes (i.e., the baits; Gnirke et al., 2009). These baits are developed from genomic resources such as whole genomes or transcriptomes (e.g., 1 KP project, Matasci et al., 2014) and can be either specific to the study group (de Sousa et al., 2014; Heyduk et al., 2016) or they capture conserved genes across higher taxonomic ranks (e.g., angiosperm bait kit, Johnson et al., 2018).

In angiosperms, most hybrid-enrichment studies opt for nuclear loci (e.g., de Sousa et al., 2014; Comer et al., 2016; Couvreur et al., 2019; Schneider et al., 2020) because of their higher sequence variation and providing unlinked, independent datasets for phylogenetic reconstruction (Small et al., 2004; Steele et al., 2008; Zimmer and Wen, 2012), but some also target the plastid genome (e.g., Faye et al., 2016; Heyduk et al., 2016; Abreu et al., 2018). Targeting both the nuclear and the plastid genome in a single sequence capture experiment is usually avoided because the plastid loci (which are present at high copy number) are enriched proportional to their presence in the genomic DNA extractions, thus generating a strong bias in favor of the plastid genome (Weitemier et al., 2014). Besides directly capturing the plastid genome by hybridization-based target enrichment using plastome specific baits, complete plastomes have also been obtained by long-range PCR (Harrison et al., 2016; Wang et al., 2018) and genome skimming (i.e., low-coverage whole genome sequencing; Straub et al., 2012). Another approach also relies on hybrid enrichment. However, instead of directly targeting the plastome, plastid sequences are indirectly captured from the off-target read fraction of a hybrid enrichment sequencing experiment (Weitemier et al., 2014; Granados Mendoza et al., 2020). Although the hereby harvested data may often permit the assembly of complete plastomes, this approach has still rarely been explored in angiosperm phylogenomics with a comprehensive taxon sampling.

The plastid genome has been traditionally widely used for the reconstruction of phylogenetic relationships at different taxonomic ranks among angiosperms because of its uniparental inheritance, high copy numbers, the generally strongly conserved structure and the blend of coding and non-coding sequence regions with highly variable substitution rates (e.g., Sun et al., 2013; Gitzendanner et al., 2018). When analyzed together with the bi-parentally inherited nuclear genome, DNA sequence data from the plastome may also provide insights into processes such as chloroplast capture, hybridization, and incomplete lineage sorting (ILS) through the detection of topological conflicts between plastid and nuclear phylogenies (Vriesendorp and Bakker, 2005; Schneider et al., 2011; Gitzendanner et al., 2018; Paule et al., 2020). In recent years, the complete plastome has even been proposed as a super-barcode because of its increasing availability and the much higher information content compared to traditional single-locus barcodes, which often do not provide resolution at the species level (Li et al., 2015). However, evolutionary rates may vary considerably between taxa (Barrett et al., 2016), compromising their utility for resolving phylogenetic relationships at lower taxonomic ranks in lineages with significant rate deceleration or recent origin (Heyduk et al., 2016). Moreover, extreme rate heterogeneity may impair phylogenomic analysis, which is aggravated by sparse taxon sampling (Philippe and Roure, 2011).

While complete plastomes become increasingly available, densely sampled phylogenomic studies of species-rich taxa based on plastid genomes are still scarce. Additionally, many angiosperm families still lack complete plastomes as, for example, the pantropical Ochnaceae. This family has been recently studied using a comprehensive taxon sampling and multiple plastid DNA sequences (Schneider et al., 2014) as well as nuclear loci obtained through targeted enrichment (Schneider et al., 2020). Ochnaceae are a family of mostly trees and shrubs, comprising about 550 species and 34 genera widely distributed and ecologically successful in tropical rain forests, dry forests, and savannas (Amaral and Bittrich, 2014; Schneider et al., 2014, 2020). They share a unique leaf venation architecture that in many genera is characterized by very densely spaced second or third order veins (Schneider et al., 2017). They are also striking for the evolution of dioecy, andromonoecy, and androdioecy in Medusygynoideae and Quiinoideae (Schneider et al., 2002; Schneider and Zizka, 2016, 2017). Major radiations in the Old and New World tropics occurred in subtribe Ochnineae which unite about two thirds of the family’s species diversity. The neotropical *Ouratea* Aubl. with its 200–310 species (Amaral and Bittrich, 2014; Schneider, unpubl. data) is by far the most species-rich genus, comparable in size to the neotropical radiations of *Inga* Mill. (ca. 300 spp., Richardson et al., 2001), *Ocotea* Aubl. (ca. 300 spp., Madriñán, 2004), *Clusia* L. (ca. 300 spp., Gustafsson et al., 2007), or *Guatteria* Ruiz and Pav. (ca. 265 spp., Erkens et al., 2007).

A first comprehensive molecular phylogenetic analysis based on five DNA sequence regions, including four from the plastid genome, resolved many parts of the Ochnaceae’s phylogenetic backbone (Schneider et al., 2014). However, uncertainty remained especially concerning relationships within Ochnineae, part of Sauvagesieae, and between the three subfamilies Ochnoideae, Medusagynoideae, and Quiinoideae. A recent taxon-rich targeted enrichment study capturing nuclear loci of more than 250 species unraveled most of the uncertain relationships, closed important taxon gaps, and revealed for the first time insights into infrageneric relationships in all of the most speciose genera of Ochnoideae (Schneider et al., 2020). Support for some of the genus-level relationships was still moderate such as for the position of *Medusagyne* Baker in relation to the other subfamilies, within the SE Asian clade of Sauvagesieae (*Euthemis* Jack, *Indosinia* J.E.Vidal, *Indovethia* Boerl., *Neckia* Korth.) and within the clade uniting the neotropical genera *Cespedesia* Goudot, *Godoya* Ruiz and Pav. and *Krukoviella* A.C.Sm. Increasing the amount of DNA sequence data by amending plastomes is likely to provide additional evidence for some of the unclear relationships and to improve the family’s phylogenetic framework that is required to tackle pending questions on the timing and pace of the major radiations of Ochnaceae in the Old and New World tropics.

In the present study, we aim at (1) corroborating the classificationsystem of Ochnaceae and providing support for hitherto poorly supported phylogenetic relationships along the family’s backbone using plastome data, (2) examining the utility of the off-target read fraction from a targeted enrichment experiment for assembling plastomes, (3) exploring the utility of the plastid genome for resolving phylogenetic relationships at different taxonomic levels using a taxon-dense, species-rich sampling strategy, and (4) assessing conflicts, if any, between nuclear and plastid genome phylogenies to uncover evolutionary processes such as organelle capture and ILS.

## Materials and Methods

### Taxon Sampling

Leaf tissue was taken from specimens of the herbaria BR, CEPEC, FR, GB, L, LZ, MO, NY, P, U, and WAG as well as from silica-dried leaves from specimens collected in the field and from a few living plants of the Royal Botanic Garden Edinburgh and the Botanischer Garten und Botanisches Museum Berlin. Initially at least one representative species of each genus following the most recent classification of Ochnaceae (Schneider et al., 2014) was included, except for the Chinese endemic *Sauvagesia rhodoleuca* (Diels) M.C.E. Amaral (= *Sinia rhodoleuca* Diels), for which material was unavailable. The initial taxon sampling comprised 282 accessions of which a maximum of 213 samples remained for phylogenetic analysis (Supplementary Table 1).

### Bait Design, DNA Extraction, Library Preparation, In-Solution Hybridization, and Sequencing

To obtain plastome data, we used the sequencing output (i.e., the off-target sequence read fraction) of a target enrichment study that captured about 660 kbp of nuclear loci as described in detail in Schneider et al. (2020). Briefly, custom baits were developed for the selected nuclear target loci based on one ingroup (*Ochna serrulata* Walp.) and four outgroup transcriptomes from the clusioid sister clade. To avoid a disproportional capture of organellar genomes, these genomes (i.e., plastid and mitochondrial) were filtered by blasting against genomic resources of *Ricinus communis* L. The final baits set targeted 275 nuclear loci that were sent off for the production of 19,398 baits with a 3.75× tiling density (each bait 120 nt long, ca. 32 nt spacing between the start of neighboring baits) at MYcroarray (now Arbor Biosciences, Ann Arbor, United States).

DNA was extracted from up to 20 mg leaf tissue. Tissue was disrupted using a Bead Ruptor 24 (Omni International, Kennesaw, United States) with three cycles at a speed of 4.2 m s^–1^ for 20 s. DNA extraction mostly followed a modified CTAB protocol (Doyle and Doyle, 1987). DNA purity was verified with a DS11 spectrophotometer (DeNovix, Wilmington, United States) and yield was measured with Qubit 1.0 (Thermo Fisher, Waltham, United States). Fragment size ranges of the gDNA samples were checked on 1% agarose gels, 2,200 TapeStation or a BioAnalyzer (Agilent, Santa Clara, United States). Based on these ranges, we assigned the samples to four groups that were subject to different protocols for DNA fragmentation: (i) highly degraded, with most fragments <0.5 kb; (ii) mostly small fragments but some faint smear up to 1 kb; (iii) smear to 2–3 kb; (iv) high molecular weight smear with fragment range up to >5–10 kb. DNA was sheared with a Bioruptor UCD 300 Next Gen sonicator (Diagenode, Liège, Belgium) at low power mode with the following parameters to achieve a fragment size range of 100–800 bp: 5 cycles of 15 s ON, 90 s OFF (group ii); 8 cycles of 20 s ON, 90 s OFF (group iii); 10 cycles 30 s ON, 90 s OFF (group iv). For highly degraded DNA (group i), sonication was omitted.

Library preparation was done with the NEBNext Ultra II DNA Library Preparation kit (New England Biolabs, Ipswich, United States) following the manufacturer’s manual. Samples were further grouped based on DNA yield (sets of 50, 100, 250, and 500 ng DNA input), to facilitate subsequent library amplification, and phylogenetic relationship (e.g., species of the same genus) to reduce potential biases during sequence capture. After adaptor ligation, the libraries were cleaned using Ampure XP beads (Beckman and Coulter, Brea, United States). This clean-up included a fragment size selection for samples with DNA input >50 ng and for groups ii-iv to achieve a mean fragment size of approximately 400 bp. In the case of lower DNA input and highly degraded DNA, size selection was usually omitted. All libraries were enriched using 5–14 PCR cycles and the reaction conditions as recommended by the manufacturer’s manual. We used a dual indexing approach with the NEBNext Multiplex Oligos for Illumina (Dual Index Primer Set 1, New England Biolabs) which provide 96 unique barcode combinations. After a final clean-up with Ampure XP beads, libraries were quantified using Qubit 1.0.

In-Solution hybridization was conducted using the MyBaits Kit (Arbor Biosciences) and the manufacturer’s protocol, version 3.0. Equimolar amounts of up to six libraries were pooled in a single hybridization reaction. For highly degraded DNA, hybridization was performed in single reactions with the baits diluted 1:2. Hybridization was run for 16–21 h at a temperature of 65°C. For some Sauvagesieae, Quiinoideae, *Medusagyne*, a hybridization temperature of 60°C was chosen following Li et al. (2013). This reduction of stringency in taxa that are phylogenetically distant from the ingroup taxon used for bait design was followed to also capture more divergent loci. Post-capture libraries were enriched during 10–14 PCR cycles using the “reamp” primers (see Meyer and Kircher, 2010; annealing temperature: 65°C) and a HiFi polymerase (KAPA HiFi HotStart Ready Mix, KAPA Biosystems, Wilmington, United States). The enriched post-capture libraries were cleaned with 1.1 × Ampure XP beads, eluted in 10 mM Tris–HCl, 0.05% Tween-20, pH 8.0, and quantified with Qubit 1.0. The pooled post-capture libraries were further pooled (up to 96 dual indexed samples) at equimolar amounts for sequencing on three Illumina HiSeq 2500 lanes and one Miseq lane with 150 bp paired-end reads at Macrogen (Seoul, South Korea). The fastq (R1 and R2) sequences for all individuals are available in Genbank SRA under the Bioproject number PRJNA602196^1^.

### Sequence Assembly

Raw reads were first filtered using BBDuk v1.0 from BBTools (Bushnell, 2019) as implemented in Geneious v11.1.5 (Kearse et al., 2012) in order to remove adapters, known Illumina artifacts, PhiX, and to quality-trim both ends to <Q20 or discard the reads if the read length was below 10 bp after trimming. Because of the lack of published plastid genomes of Ochnaceae, we chose a plastome from the clusioid sister clade as a reference. The reads of each sample were mapped to the edited plastome of *Garcinia mangostana* L. (131,170 bp; GenBank accession NC_036341.1; Jo et al., 2017) containing one inverted repeat (IR) only using the Geneious mapper, custom sensitivity (gaps per read 5%, maximum gap size five, word length 20, index word length 15, maximum mismatches per read 10%, maximum ambiguity four, ignoring words repeated more than 20 times) and 25 iterations. In this way, we could identify a set of samples with the highest numbers of plastome reads as well as the highest coverage of the *Garcinia* plastome.

Using Fast-Plast v1.2.8 (McKain and Wilson, 2018) raw data of 10 selected samples with high read numbers and from across most of the major clades of Ochnaceae (*Brackenridgea arenaria* (De Wild. and T. Durand) N. Robson, *Campylospermum obtusifolium* (Lam.) Tiegh., *Campylospermum vogelii* (Hook.f. ex Planch.) Farron, *Luxemburgia diciliata* Dwyer, *Medusagyne oppositifolia* Baker, *Ochna integerrima* (Lour.) Merr., *Ouratea bahiensis* Sastre, *Poecilandra pumila* Steyerm., *Sauvagesia glandulosa* (A. St.-Hil.) Sastre, *Sauvagesia nitida* Zappi and E. Lucas) were analyzed in order to generate *de novo* plastomes. The pipeline first cleaned the reads utilizing Trimmomatic v0.39 (Bolger et al., 2014) and extracted plastid-derived reads with Bowtie v2.3.5.1 (Langmead and Salzberg, 2012), using the Malpighiales taxa included as references in Fast-Plast. The reads were *de novo* assembled by the combination of the De Bruijn graph-based method of SPAdes v3.14.0 (Bankevich et al., 2012) with an iterative seed-based microassembly implemented in afin^2^, which tries to close gaps of contigs with low coverage. If no single contig was obtained, SSPACE v2.1.1 (Boetzer et al., 2010) was used for scaffolding. Unfortunately none of the *de novo* analyses yielded a full plastome assembly (i.e., none of these assembled contigs exceeded 79 kbp except *Ouratea bahiensis*). Hence, for further analyses the longest recovered contig for an Ochnaceae species (138,408 bp, *Ouratea bahiensis*) was annotated using GeSeq (Tillich et al., 2017) and shortened to remove the duplicated IR fragment. This led to a reference sequence of 100,891 bp which comprised the IR, the small single-copy (SSC) and part of the large-single-copy (LSC) region including 55 CDS, 24 exons, 18 tRNA (the annotated reference contigs before and after removing duplicated IR fragments are available as Supplementary Data 1). Subsequently, reads of each sample were mapped to this reference using the Geneious mapper, applying the settings mentioned above. Majority consensus sequences were called with a minimum coverage of 3× (dataset 1) and 2× (dataset 2). We only kept samples matching at least 45% of the reference sequence at given coverage to avoid issues with excessive missing data.

### Phylogenetic Reconstruction and Statistics

Both datasets were aligned with MAFFT v7.470 (Katoh and Standley, 2013) using default parameters and automatic selection of the appropriate alignment strategy. Maximum likelihood (ML) phylogenetic analyses were performed using RAxML-HPC BlackBox v8.2.12 (Stamatakis, 2014) on CIPRES Science Gateway v3.3 (Miller et al., 2010) and XSEDE (Towns et al., 2014) using default settings and automatic bootstrapping halt. We also ran the phylogenetic analyses after trimming the alignment with trimAl v1.2rev59 (Capella-Gutierrez et al., 2009) using the implemented heuristic method for an automated determination of the best trimming method (here, the “gappyout” method). For all subsequent analyses, we used the DNA sequence data without further partitioning. Omitting data partitioning was based on the rationale that tree topologies inferred from ML analyses under various partitioning schemes remained mostly the same as in the unpartitioned analysis as reviewed by Ma et al. (2014). Although partitioning of plastomes can provide a better fit to the data, a strong phylogenetic signal at lower taxonomic levels can robustly resolve the relationships even without the use of an optimal partitioning scheme (Pham et al., 2017; Dillenberger et al., 2018; Paule et al., 2020).

For a comparison of phylogenetic reconstructions of plastome versus nuclear loci, we included the nuclear trees obtained during a previous target enrichment study based on the same set of Ochnaceae samples and raw sequence data (Schneider et al., 2020). We used the R package phytools v0.6-99 (Revell, 2012) and the “cophylo” function to draw co-phylogenetic plots of both genomic datasets. For the nuclear loci, this was done for each major clade separately based on the approach followed in Schneider et al. (2020): the concatenated alignments of the nuclear loci comprised 143,406 nucleotides for Ochneae (therein called LEO dataset) and 58,319 nucleotides for Luxemburgieae, Sauvagesiae, and Testuleeae (therein called SLT dataset). We used the trees obtained from the analysis with concatenated data because the topologies were largely congruent with those obtained from the multispecies coalescence approach (Schneider et al., 2020).

Trees were processed with the packages ape v5.3 (Paradis and Schliep, 2018) andggtree v2.0.1 (Yu et al., 2017) in R, v3.6.2 (R Core Team, 2019). Pearson correlation analysis for the relationship between the proportion of recovered plastid nucleotides and the number of cleaned sequence reads that map to the plastid genome was conducted using the R package ggpubr v0.2.4. The numbers of variable and parsimony informative sites were calculated with AMAS v0.98 (Borowiec, 2016).

## Results

### Assembly, Dataset, and Alignment Statistics

We analyzed raw sequence data from 282 Ochnaceae samples and oneoutgroup [*Bonnetia stricta* (Nees) Nees and Mart.]. The plastid sequences were recovered from the cleaned off-target read fraction of the targeted enrichment of nuclear loci and varied considerably in number. Concerning the *G. mangostana* reference, 82 to 373,197 reads per sample with 0.08 to 374× mean coverage were mapped matching 3.2 to 97.9% of the reference sequence. A total of 292 to 352,928 cleaned reads per sample were mapped to the “*de novo* contig” reference (Supplementary Table 2) with 0.26 to 496× mean coverage matching 5.7 to 100% of the reference sequence. Based on our quality threshold, subsets of 181 samples in dataset 1 and 213 samples in dataset 2 were kept (Supplementary Tables 1, 2). Final alignments comprised 116,191 and 117,923 bp (101,007 bp after trimming with trimAl) for datasets 1 and 2, respectively (Table 1 and Supplementary Data 2–4).

**TABLE 1:** Assembly and alignment characteristics of the two plastome datasets used for the phylogenetic analysis after alignment trimming and filtering (including dataset two after automated trimming with trimAl).

	Dataset		
	1	2	2 (trimAl)
Number of species (samples)	171 (181)	201 (213)	201 (213)
Mean (and range of) number of nucleotides (bp)	90,282 (46,983–100,901)	88,844 (47,267–100,908)	n.d.
Alignment length (bp)	116,191	117,923	101,007
Number of variable (parsimony informative) sites	36,993 (18,204)	41,153 (19,908)	37,363 (18,812)

**FIGURE 1:**
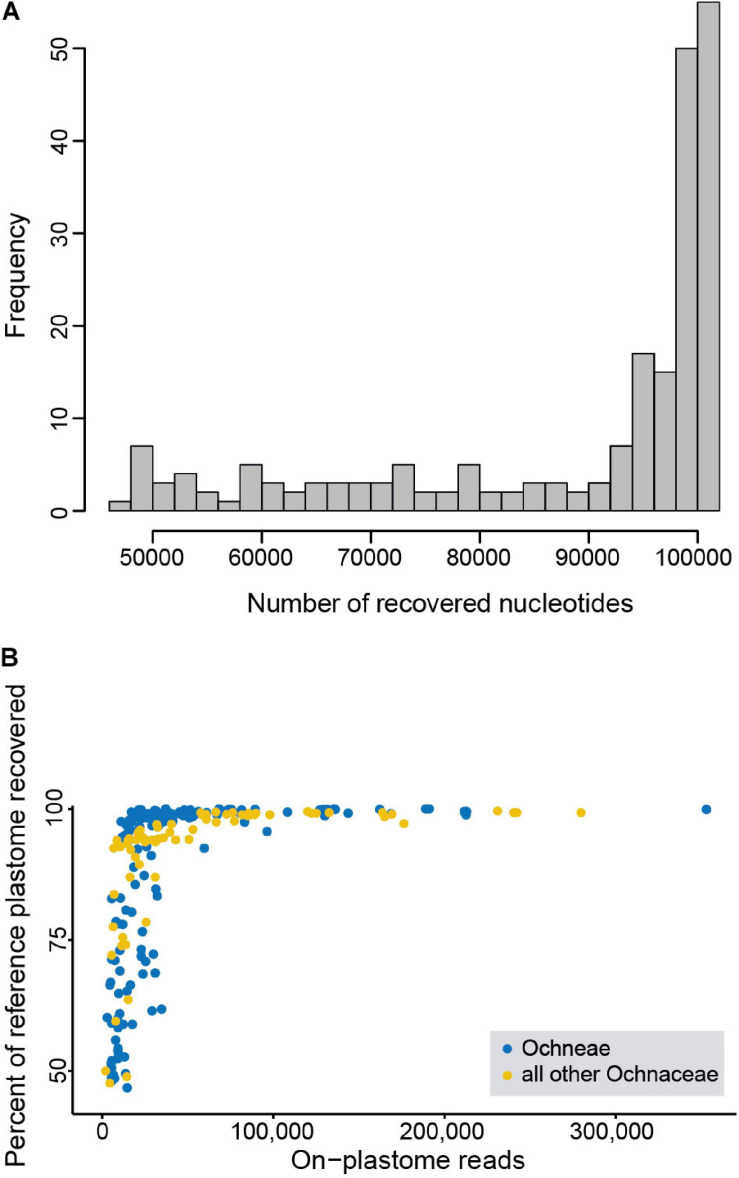
Recovery of plastomes from targeted enrichment. **(A)** Amount of the plastome recovered (i.e., number of nucleotides) across Ochnaceae in relation to the reference plastome (based on dataset 2). **(B)** Percentage of the reference plastome recovered in relation to the number of sequence reads that map to the reference (based on dataset 2).

For the reads mapping to the plastome, the full length of the*de novo* contig reference plastome was usually consistently obtained with more than 50,000 reads. In some samples it was also achieved with less reads, but mostly only a fraction of the reference was recovered in such cases (Figure 1 and Supplementary Table 2). On average, about 2% of the cleaned reads mapped to the plastid genome. The number of nucleotides (i.e., the un-gapped sequence length) of the plastome recovered per sample ranged between 46,983 and 100,901 (mean: 90,282) for dataset 1; and between 47,267 and 100,908 for dataset 2 (mean: 88,844 bp; Supplementary Table 2). This translates into mean proportions recovered from the reference plastomes of 89.5 and 88.1% for both datasets (Supplementary Table 2). For dataset 2, for example, 121 samples (i.e., 56.5% of the samples) recovered >95% of the reference plastome. For dataset 2, we also compared the number of variable and parsimony informative sites of the most species-rich genus of each of the tribes of Ochneae (except the monotypic Testuleeae). In *Luxemburgia* A.St.-Hil., *Ouratea* and *Sauvagesia* L. we observed 3,241, 11,675, and 12,893 variable sites, respectively, of which 774, 2,638, and 5,561 were parsimony informative.

### Phylogenetic Relationships of Ochnaceae

**FIGURE 2:**
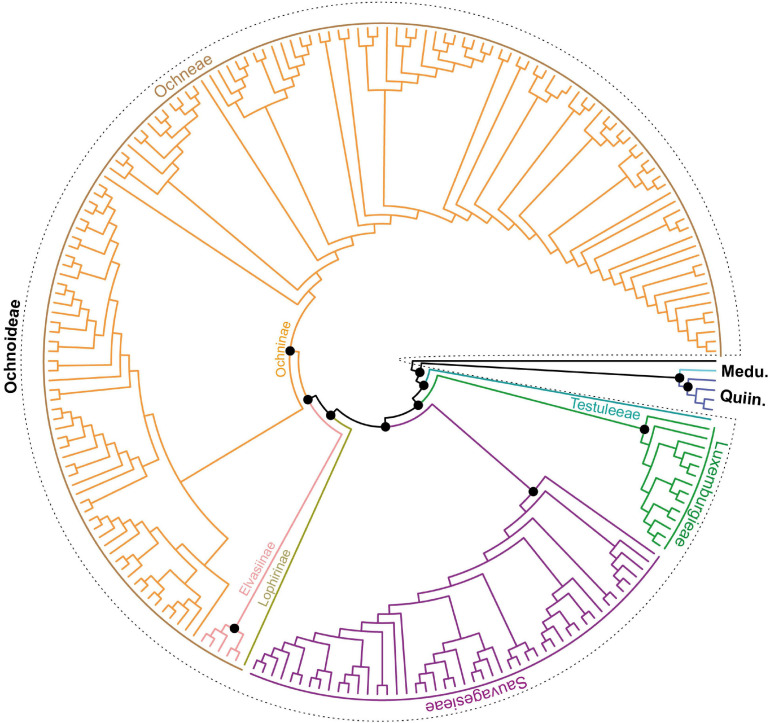
Overview of the phylogenetic relationships of the major clades of Ochnaceae based on RAxML analysis of plastome dataset 2 and 213 accessions. Bootstrap support is given for all backbone nodes including all major clades (black circle = 100% BS). The classification follows Schneider et al. (2014). Ochninae is by far the most species-rich clade comprising about two thirds of the family’s species and six genera. Quiin., Quiinoideae; Medu., Medusagynoideae.

**FIGURE 3:**
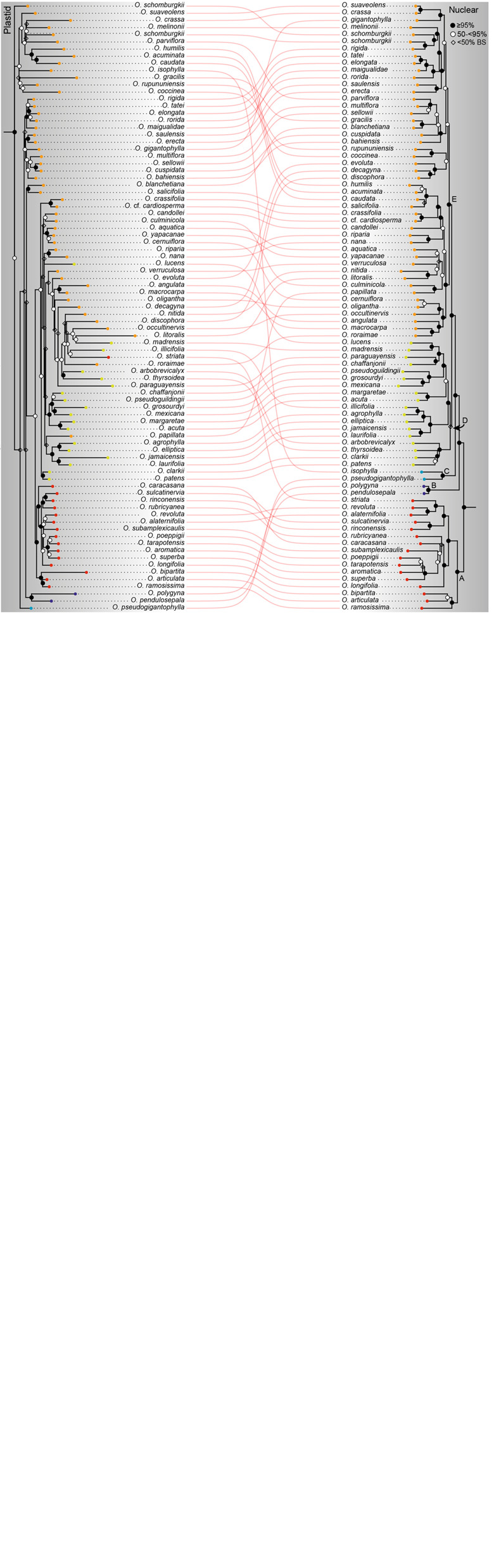
Co-phylogeny plot of plastome and nuclear phylogenies of Ochnaceae from targeted enrichment. The plastome tree is based on dataset 2, the nuclear phylogeny is taken from Schneider et al. (2020). Conflict between trees is indicated by branch swaps as illustrated by the interconnecting lines that link identical plant accessions. Lack of interconnecting lines indicates taxa not present in the complementary tree. Bootstrap support is grouped by range and shown as black or open circles at the internal nodes (diamond as a third symbol for BS <50% in *Ouratea* only). Colored circles at tips indicate major clades. A to D refer to clades of *Ochna* retrieved in Schneider et al. (2020). A dashed line links the topology of the relationships of the subfamilies of Ochnaceae with the topology of Ochnoideae.

The phylogenetic reconstructions from both assembly approaches support similar topologies (Figures 2, 3 and Supplementary Figures 1–3), except for the differences arising from uneven taxon sampling and few relationships as described below. Therefore, we mainly refer to the phylogenetic tree with the most complete taxon sampling, i.e., from dataset 2, and without automated trimming because of minor differences in branch support only (see below).

The plastome tree unraveled the phylogenetic relationships at the deep nodes of Ochnaceae, providing for the first time maximum support for the hitherto recalcitrant *Medusagyne* as sister to Quiinoideae (Figures 2, 3). Within Quiinoideae, *Lacunaria* Ducke and *Touroulia* Aubl. are sister to *Quiina* Aubl., all three being sister to *Froesia* Pires (all receiving maximum support). Ochnoideae were recovered as a monophyletic subfamily (100% BS). Therein, Ochneae and Sauvagesieae are sister clades (each with 100% BS), both being sister to Luxemburgieae, and all three tribes together are sister to the monotypic Testuleeae (all with 100% BS). Luxemburgieae were recovered as monophyletic with maximum support including its two genera *Philacra* Dwyer and *Luxemburgia*. *Luxemburgia glazioviana* Beauverd turned out to be the sister to the remaining species of the genus, the latter falling into two strongly supported major clades.

Among Sauvagesieae, the non-monophyletic *Sauvagesia* falls into two separate clades. Clade A is sister to clade B plus *Adenarake* Maguire and Wurdack (all with 100% BS). Backbone nodes of *Sauvagesia* all receive strong support. Only a few interspecific relationships of clade A are weakly supported. *Tyleria* Gleason was recovered as sister to *Sauvagesia* with strong BS, both clades being sister to a clade uniting all SE Asian species of this tribe (*Neckia*, *Schuurmansiella* Hallier f. and allies; 100% BS). Altogether, they are sister to *Poecilandra* Tul. and *Wallacea* Spruce ex Benth. and Hook. f. (100% BS). The basal-most node divides Sauvagesieae into *Blastemanthus* Planch. and the moderately supported clade (88% BS) that comprises the previously described taxa and its sister clade uniting *Cespedesia*, *Fleurydora* A.Chev., *Godoya*, *Krukoviella*, and *Rhytidanthera* Tiegh. In the tribe Ochneae, Ochninae and Elvasiinae form a sister group, both being sister to *Lophira* Banks ex C.F.Gaertn. (all with 100% BS). Elvasiinae comprises the two genera *Elvasia* DC. and *Perissocarpa* Steyerm. and Maguire (100% BS). *Ouratea* is sister to the rest of Ochninae (99% BS). A clade of *Ochna* L. and *Campylospermum* Tiegh. clade A is sister to the remaining three genera of Ochninae (100% BS). Within the latter, *Idertia* (Oliv.) Farron is sister to a clade of *Campylospermum* clade B and *Brackenridgea* A.Gray (100% BS).

Dataset 1 produced a highly similar topology compared to dataset 2 with a largely identical backbone of the family. Trimming gappy sites from the alignment also did not alter significantly the phylogenetic inferences. It mainly led to some slight reductions in branch support (e.g., *Testulea* Pellegr. as sister to the rest of Ochnoideae received only 98% BS instead of maximum support).

### Comparison of Plastid and Nuclear Topologies

Phylogenetic conflict between the plastome and nuclear topologies is generally low and largely confined to interspecific relationships of the rapidly diverging genera of Ochninae, which is generally associated with weak or moderate branch support in the plastome tree. A major well-supported swap is observed in *Campylospermum*: In the nuclear tree, *Campylospermum* clade A is sister to *Rhabdophyllum* Tiegh., whereas in the plastome tree, it is sister to *Ochna* (but *Rhabdophyllum* is lacking in that tree). In Sauvagesieae, a well-supported conflict is observed, for example, in *Sauvagesia*, with *S. tenella* Lam. as sister to a clade of *S. longifolia* Eichler and *S. elata* Benth. with maximum support. However, in the nuclear species tree of Schneider et al. (2020), the clade of *S. longifolia* and *S. elata* is sister to a clade of seven species, with *S. tenella* nested in between. Additionally, the position of *S. erecta* L. switched. In the nuclear phylogenetic tree, this species was sister to a clade of six species, whereas in the plastome tree it was nested together with *S. brownii* Planch. in the same set of species (but without *S. tenella*). In *Campylospermum*, the infrageneric relationships largely agree between both the plastome and nuclear trees. Only in clade A, there is a well-supported conflict regarding the sister group relationship of *C. duparquetianum* Tiegh. In the plastome tree, this species is sister to a *C. sulcatum* (Tiegh.) Farron, whereas in the nuclear tree, it is sister to *C. congestum* (Oliv.) Farron. In both trees, each of these clades is sister to the rest of the clade A.

## Discussion

### Plastome Recovery From Targeted Enrichment of Nuclear Loci

In the present study, we explored the potential of recovering plastomes from targeted enrichment designed to capture nuclear loci. Such targeted enrichment produces a considerable amount of sequencing reads that do not match the selected target loci. This fraction of off-target reads also contains, among others, reads that match the plastid genome, despite filtering plastid loci during bait design (Weitemier et al., 2014). In this study, the off-target fraction comprised up to more than 10 million reads per sample. However, the proportion of reads that map to the plastid genome was rather small with a maximum of about 360,000 reads. With about 50,000 or more on-plastome reads, our *de novo* reference plastome was usually entirely recovered (Figure 1). On the other hand, we did not obtain an entire plastome with *de novo* assembly, although only a relatively moderate number of reads is required to cover an estimated mean plastome length of 150 kb. Obviously, some portions of the plastome were underrepresented at the given quality threshold (minimum coverage 3×) even in the samples with the highest proportion of the plastome fraction. The longest available contig was obtained for *Ouratea bahiensis* (ca. 138 kbp), which is however in the range of plastome lengths observed among Malpighiales (185 spp. with ca. 130–170 kb; GenBank, accessed 17 Feb 2020; Bedoya et al., 2019). One reason for lower or insufficient coverage is that part of the samples was sequenced with lower output during an initial test run (Schneider et al., 2020). Another cause of the incomplete recovery of the plastomes is the distance from the reference plastome (here, from *Ouratea bahiensis*), which will often affect the assembly of structural variants and of the more rapidly evolving non-coding regions (Straub et al., 2012), including inversions. An additional effect may come from the efficiency of the targeted enrichment: the lower the enrichment of the nuclear targets the higher the proportion of off-target reads and, thus, of reads that match the plastome. Furthermore, the method of preservation of the tissues used for DNA extraction influences the assembly success, with plastomes being more difficult to assemble for samples derived from herbarium specimens that have been initially sprayed with alcohol while collected in the field and before the drying process. Such specimens were also used in this study and usually show more contigs with lower N50 values due to stronger degradation of the DNA (Bakker, 2017).

Among the taxa with low mean coverage and contig lengths, several were filtered based on our threshold set for the amount of missing data. During sequence assembly, we opted for two approaches to find a balance between the number of recovered nucleotides (i.e., contig length), taxon sampling, and data quality (in terms of primary sequence errors). This included the lowering of the minimum coverage threshold below the default in dataset 2 (2×) The lower threshold led to increased contig lengths in several samples, which also increased the number of taxa available for phylogenetic analysis, for example from 181 in dataset 1 to 213 in dataset 2. The downside of lowering the coverage threshold is that the risk of incorporating primary sequence errors (here, assembly errors) increases and, thus, of non-phylogenetic signal in the low-coverage regions of the contigs. Such errors can be more detrimental to phylogenetic inference than alignment errors (di Franco et al., 2019). However, a positive effect comes from avoiding issues from incomplete taxon sampling, which can also lead to inaccurate phylogenetic inference when associated, for example, with long branches (Heath et al., 2008; Wiens and Tiu, 2012; Streicher et al., 2016). Comparing the phylogenetic trees obtained from datasets 1 and 2, we did not find significant well-supported differences in topology along the backbone of Ochnaceae. On average, the more taxon-rich dataset 2 reconstructs the phylogenetic relationships with higher BS relative to dataset 1 (except for *Ouratea*) which has fewer taxa but was assembled with the default minimum coverage. Dataset 2 also generated a phylogenetic tree that is more similar to the nuclear tree in Schneider et al. (2020). Although the comparison of topologies based on the different consensus calling strategies together with the congruence of the previously published nuclear tree topology does not provide direct evidence for the influence of primary sequence errors, the similar positioning across the phylogenetic trees of the taxa subjected to the lowered coverage threshold suggests that such errors only marginally affect the plastome-based phylogenetic inference in dataset 2 and that they are most likely outcompeted by the positive effect arising from filling important taxon gaps. An inaccurate phylogenetic inference may also arise from alignment errors, such as erroneous homology statements or the saturation of substitutions, often associated with highly variable alignment positions (Philippe et al., 2011). Alignment trimming is a widely used remedy to remove problematic positions. In this study, we examined the effects of alignment trimming on the phylogenetic reconstruction using dataset 2 as an example. The automated trimming reduced the length of the alignment by about 15%. However, differences in reconstructed phylogenetic relationships between both the trimmed and untrimmed alignments were minor, except for a drop in branch support at infrageneric level in Ochninae in the first. Thus, the trimming apparently removed data that informed the relationships at a shallow level, which indicates that the data removal might have increased stochastic error and decreased phylogenetic accuracy (di Franco et al., 2019).

### Phylogenetic Resolution at Deep and Shallow Levels

The phylogenetic backbone of Ochnaceae was largely resolved with the plastome data, similar to the relationships inferred from the nuclear loci of the same targeted enrichment experiment (Schneider et al., 2020), thus providing additional evidence for the classification system of Ochnaceae established in Schneider et al. (2014). The plastome data resolve the relationships among the three subfamilies for the first time with maximum branch support, corroborating the earlier hypothesized sister group relationship of Quiinoideae and the monotypic Medusagynoideae (Xi et al., 2012; Schneider et al., 2014, 2020), which are both sister to Ochnoideae. This relationship has long been unclear due to shifting positions and poor support (e.g., Fay et al., 1997; Savolainen et al., 2000; Davis and Chase, 2004). Such recalcitrant clades are notoriously difficult to resolve because of their (rapid) divergence at a deep time combined with long stem branches (King and Rokas, 2017) and are often intractable even with high amounts of data (Francis and Canfield, 2020). In the present study, both datasets with different taxon sampling and amount of nucleotides recovered these subfamilial relationships with maximum support, even after automated trimming, so we are confident that this evidence is fairly robust and not based on few informative sites that exert a strong influence on the topology as observed even in data-rich phylogenomic analyses (Shen et al., 2017; Francis and Canfield, 2020).

The four tribes of Ochnoideae – Luxemburgieae, Ochneae, Sauvagesieae, and Testuleeae – were retrieved with maximum support with both datasets of the plastome data (Testuleeae not present in dataset 1), which is also an improvement compared to the nuclear data. With the latter, the same relationships were retrieved, but the clade of Ochneae and Sauvagesieae received strong support only with one dataset (Schneider et al., 2020). With the more clade-specific dataset used for comparison with the plastome data, support was moderate (Figure 3), which however might be an artifact from high amounts of missing data in Ochneae in this nuclear dataset (Schneider et al., 2020).

The plastome data also corroborate the sister group relationship of *Luxemburgia* and *Philacra* (the latter was filtered for the nuclear data based on the amount of missing data in Schneider et al., 2020) already established in Schneider et al. (2014), which is also backed by flowers that are obliquely zygomorphic already in the bud with the stamens surrounding the ovary only adaxially and filaments that are basally or completely fused (Amaral, 1991; Amaral and Bittrich, 2014; Schneider et al., 2014). Both genera are largely confined to open rocky savannas, with *Luxemburgia* having diversified especially in the mountaintops of the Espinhaço Range of Eastern Brazil (Amaral, 1991; Feres, 2001, 2007, 2010). Earlier attempts to resolve the interspecific relationships of this genus using the internal transcribed spacer region (Feres, 2001) or nuclear loci from targeted enrichment (Schneider et al., 2020) suffered from weak branch support for most of its internal branches. The plastome data of the present study improved the phylogenetic framework for this genus, thereby permitting the evaluation of Beauverd’s (1915; also followed by Dwyer, 1951 and Feres, 2001) infrageneric classification that divided this genus into sections *Petiolatae* Beauverd and *Epetiolatae* Beauverd, based on whether species are distinctly petiolate or not. Our molecular data confirmed the petiolate *Luxemburgia glazioviana*, an endemic species of rocky outcrop vegetation on the inselbergs surrounded by Atlantic montane rain forest in Rio de Janeiro, as sister to the rest of the genus (Figure 2; Schneider et al., 2020). It is noteworthy that all other *Luxemburgia* species sampled here are mostly narrow endemics of the campos rupestres vegetation on the mountaintops scattered across the Espinhaço Range in Minas Gerais (Feres, 2001, 2010). However, infrageneric clades unite species with petiolate and sessile leaves, as for example the clade of *Luxemburgia ciliatibracteata* Sastre, *L. schwackeana* Taub. (both sessile), *L. damazioana* Beauverd and *L. ciliosa* Gardner (both petiolate), making the sections non-monophyletic and calling for a revision of the diagnostically relevant characters that may support the presented data here.

In Sauvagesieae, differences between the plastome and nuclear trees are minor. Perhaps most remarkable is the strongly supported position of *Neckia* as sister to the rest of the Asian species of that tribe in the plastome tree. This contradicts the findings from the nuclear data in Schneider et al. (2020) in which *Neckia* came out as sister to *Indosinia*, albeit with moderate support only. In an earlier study (Schneider et al., 2014), *Neckia* was retrieved as sister to a weakly supported clade uniting the remaining Asian species, plus *Tyleria*, *Sauvagesia*, and *Adenarake*, but based on much lower taxon and molecular sampling. The plastome tree also recovered a well-supported clade of *Euthemis*, *Indosinia*, and *Indovethia*, which are sister to *Schuurmansia* Blume and *Schuurmansiella*. In the nuclear tree, the last two were sister to the rest of the Asian species. Overall, branch support for the relationships among the Asian genera was highest in the nuclear tree of Schneider et al. (2020). However, uncertainty remains even after adding the plastome data, as shown by the shifting positions and the persistence of poor support for some of their nodes. Among the early diverging nodes of Sauvagesieae, there is also discord regarding the position of *Blastemanthus*. In the plastome tree, this genus is inferred as sister to the rest of the tribe, whereas in the nuclear tree it is sister to *Fleurydora* plus the neotropical clade of *Rhytidanthera* and allies, each with strong support. The position of *Blastemanthus* in the plastome tree was also retrieved in Schneider et al. (2014). However, the conflicting topologies highlight that the relationship of *Blastemanthus* remains uncertain.

The plastome data add further evidence to remove *Indovethia* and *Neckia* from the synonymy of *Sauvagesia*, in which both were sunk according to a concept that was originally established and expanded by Sastre (1971) and Amaral (1991, 2006). Re-erection of *Neckia* was already suggested by Schneider et al. (2014) based on their molecular data. Schneider et al. (2020) demonstrated that *Indovethia* was also independent from core *Sauvagesia*. Actually, the monotypic *Sinia* Diels is the only remaining Asian member still in the synonymy of *Sauvagesia* because it has not been available for our molecular studies to date. If *Sinia* were also independent, then *Sauvagesia* would be essentially neotropical [except for the African *S. africana* (Baill.) Bamps and the pantropical weed *S. erecta*].

Core *Sauvagesia* is divided into two major clades, both in the plastome and nuclear trees. Clade B, which contains the type species of *Sauvagesia*, is sister to *Adenarake*, the latter making *Sauvagesia* non-monophyletic. Solutions to this problem are either to sink *Adenarake* into *Sauvagesia* or to re-erect a genus for clade A, choosing among names available from the synonymy of *Sauvagesia* (e.g., *Lavradia* Vell.). The pros and cons of each scenario were already discussed in detail in Schneider et al. (2020). A decision on which scenario is, however, postponed until a modern revision of *Sauvagesia* becomes available. Interestingly, the relationships within each subclade of this genus are remarkably well resolved, far better than the infrageneric relationships in Ochninae (see below). The clade A of *Sauvagesia*, just like a subclade of *Luxemburgia* recovered here, represents another replicated radiation of Ochnaceae that diversified and remained largely endemic to the campos rupestres highlands of the Espinhaço Range.

For Ochneae, all comprehensive molecular studies to date (Bissiengou, 2014; Schneider et al., 2014, 2020), including the present study, retrieved *Lophira* (i.e., Lophirinae) as sister to a clade of Elvasiinae and Ochninae. Thus, an earlier morphology-based hypothesis that *Lophira* belongs in Sauvagesieae (Kanis, 1968) is unanimously rejected (for a detailed discussion on morphological evidence see Schneider et al., 2014).

By far the most species-rich subtribe Ochninae contains six genera, among which the three largest genera (*Campylospermum*, *Ochna*, and *Ouratea*) unite almost two thirds of the species diversity of the family. Milestones achieved by the recent molecular phylogenetic studies were (i) the discovery of the polyphyly of *Campylospermum* which was first observed in Bissiengou (2014) and corroborated by Schneider et al. (2020) and the present study; (ii) the confirmation of monophyly of the remaining genera with large taxon sampling using the plastome (present study) and nuclear data (Schneider et al., 2020); and (iii) the resolution of the backbone of Ochninae based on the nuclear loci in Schneider et al. (2020).

The infrageneric relationships within Ochninae have remained difficult to resolve most likely because of the split of the genera over a short period with subsequent rapid radiations (Schneider et al., 2017, 2020). The plastome data of the present study resolved only part of the backbone of this subtribe, with moderate support for the clade of the palaeotropical genera (all but *Ouratea*) and for the clade uniting *Brackenridgea*, *Campylospermum* clade B, and the monotypic *Idertia*. A major oddity of the plastome tree is the recovery of *Campylospermum* clade A as sister to *Ochna* with strong support. This contradicts the findings based on the nuclear data which strongly support *Campylospermum* clade A as sister to *Rhabdophyllum* – which is also supported by morphology (Schneider et al., 2020) – and *Ochna* as sister to a clade of *Brackenridgea*, *Campylospermum* clade B, and *Idertia*. The reason for this odd placement is perhaps the lack of *Rhabdophyllum* in the plastome tree. Although not an issue of long branches, incomplete taxon sampling as in the case of *Rhabdophyllum* may lead to inaccurate phylogenetic results when, for example, branch lengths are mis-estimated (Heath et al., 2008). Within *Brackenridgea*, the plastome data support the same relationships as the nuclear loci (Schneider et al., 2020), with the two major clades uniting the Afro-Malagasy and the Asian-Australian species each.

The confirmation of two phylogenetically separate clades in the non-monophyletic *Campylospermum* again calls for an updated revision of this genus. Clade A unites all Central and West African species of this genus except for *C. elongatum* (Oliv.) Tiegh., whereas clade B contains the Malagasy (here, only *C. obtusifolium* included) and East African species plus *C. elongatum*. There is also some evidence from morphology for both clades (Bissiengou, 2014; Schneider et al., 2020), however, further investigation is required to find additional characters in support of these clades.

Concerning the three largest genera, the plastome data recover infrageneric relationships with lower branch support on average compared to the nuclear data. This is especially evident in *Ouratea*, for which Schneider et al. (2020) had tentatively delimited five major clades, all but one with maximum branch support. In the plastome tree, most of the phylogenetic backbone of *Ouratea* received poor support, and only clades A and B were well-supported. Clades D and E were poorly recovered and appear intermingled, while clade A is nested within them. However, this scenario received stronger support from the dataset 1. Thus, there is a strong discord concerning the composition and relationships among the major clades of this genus when compared to the nuclear topology, a conflict that is also difficult to resolve from a morphological perspective. Neither the nuclear, nor the plastome data infer relationships as expected according to the actual infrageneric classification of Sastre (1995). So, there is a clear need for re-evaluating the infrageneric classification and the diagnostically important morphological characters. As already outlined in Schneider et al. (2020), it might be difficult to find such character combinations for the major clades in view of the short branches during the early divergence of the major clades, creating a scenario of ILS at deeper time (Xu and Yang, 2016) and many clades with not yet well fixed traits. In *Ochna*, which is the second most species-rich genus of the family, clades A and B of the nuclear data were retrieved in similar positions on the plastome tree, but clades C and D appeared as a single clade with the majority of the shallower nodes receiving weak to moderate support. However, Clades C and D each corresponded to sections of Robson’s (1963) infrageneric classification, providing support from morphology for the phylogenetic relationships derived from the nuclear data.

To sum up, the plastome data provided novel insights into the phylogenetic relationships in Ochnaceae by increasing support for deep nodes at subfamilial level, by the addition of new taxa compared to the analysis of the nuclear loci, and by supporting alternative hypothesis for the relationships among the Asian genera of Sauvagesieae. At shallow levels, these data provided for example, increased phylogenetic resolution in *Luxemburgia* compared to the nuclear data, but far less so for the species-rich genera of Ochninae. We might suspect that plastomes were better recovered and had more potentially informative data on average for species other than Ochneae, which were generally less effective in capturing nuclear loci during the present targeted enrichment experiment (Schneider et al., 2020), therefore providing higher amounts of off-target reads for plastome mapping. However, the proportion of the reference plastome recovered during read mapping does not differ significantly between Ochneae and the remaining Ochnaceae (Figure 1). It is rather the higher amount of potentially phylogenetically informative sites per species which most likely led to an increased phylogenetic resolution, as observed in the most species-rich genera of Luxemburgieae and Sauvagesieae compared to Ochneae. In *Sauvagesia*, higher substitution rates might be associated with shorter generation time (Smith and Donoghue, 2008) because it is the only genus with mostly herbaceous species. On the other hand, low infrageneric resolution due to insufficient variability even with complete plastome data is not uncommon among angiosperms as observed in young radiations or in clades with decreased substitution rates (Barrett et al., 2016; Harrison et al., 2016; Heyduk et al., 2016; Heckenhauer et al., 2019; Huang et al., 2019).

Remarkably, topological conflict between the plastome and nuclear data is scarce. The few well-supported discordances between them, in particular in *Sauvagesia* and *Campylospermum*, may be indicative of hybridization and chloroplast capture as observed in other plant groups (e.g., Schneider et al., 2011; Folk et al., 2017), the first perhaps being far more frequent in tropical woody plants than originally assumed (Schley et al., 2020). ILS might be also invoked as a likely source of conflict as was done for the analysis of the nuclear data in Ochnaceae (Schneider et al., 2020). However, other factors that recently have gained increased attention cannot be ruled out as generating conflict, as for example stochastic effects from sites with low information content (Shen et al., 2017; Walker et al., 2018, 2019; Zhang et al., 2020) or heteroplasmic recombination (Sancho et al., 2018). In the largest genus *Ouratea*, there is little well-supported conflict, but the overall resolution is very low despite a sufficient amount of phylogenetically informative sites. This might show some conflict even within the plastome data as recently highlighted by other studies (Walker et al., 2019; Zhang et al., 2020). An in-depth analysis of these factors in the taxa supposedly involved in these processes might be worth performing to better understand the contribution of each of them, particularly for the diversification of the species-rich genera in Ochnaceae.

## Conclusion

Our study successfully harvested the off-target sequence read fraction for the assembly of (partial) plastid genomes in more than 200 species of Ochnaceae. The plastome data corroborated most of the phylogenetic backbone of Ochnaceae, but provided far lower phylogenetic resolution in the species-rich Ochninae than the nuclear loci from the same targeted enrichment study, in particular at a shallow level. Overall, topological conflict was remarkably low between both the plastid and nuclear genome datasets, although a species-level comparison was hampered due to the low phylogenetic resolution in the two largest genera (*Ouratea* and *Ochna*) in the plastome tree. The few instances of well-supported discord among Ochninae may be attributed to issues of taxon sampling, including the swap of *Campylospermum* clade A to a position as sister to *Ochna*. While less informative at shallow levels, the plastome data helped to strengthen the classification of Ochnaceae, leaving few open questions regarding intergeneric relationships. Most importantly, future studies should aim at including *Sinia* (= *S. rhodoleuca*) to clarify the circumscription of *Sauvagesia*, i.e., whether it includes any Asian representative or whether all of them form independent lineages as confirmed for the other two representatives to date. This study also sets the stage for further revising the taxonomy of two non-monophyletic genera so as to make the necessary nomenclatural changes. The combined plastome and nuclear data provide an important baseline for exploring the historical biogeography and the dynamics of the major radiations of Ochnaceae.

## Data Availability Statement

The data presented in the study are deposited in GenBank SRA under the Bioproject number PRJNA602196 (http://www.ncbi.nlm.nih.gov/bioproject/602196).

## Author Contributions

JS, JP, TB, and GZ designed the research. JS performed the molecular experiments. JP, JS, and TJ carried out the data analyses. JS, DC, and AA contributed to the collection of plant material. JS and JP drafted the manuscript with contributions from all authors.

## Conflict of Interest

The authors declare that the research was conducted in the absence of any commercial or financial relationships that could be construed as a potential conflict of interest.

## Footnotes

1 http://www.ncbi.nlm.nih.gov/bioproject/602196

2 https://github.com/afinit/afin/

## Supplementary Material

Supplementary Figure 1 Maximum likelihood tree of Ochnaceae based on RAxML analysis and plastome dataset 2 (without trimming).

Supplementary Figure 2 Maximum likelihood tree of Ochnaceae based on RAxML analysis and plastome dataset 2 after automated trimming with trimAl.

Supplementary Figure 3 Maximum likelihood tree of Ochnaceae based on RAxML analysis and plastome dataset 1.

Supplementary Table 1 List of specimens with voucher information.

Supplementary Table 2 Assembly statistics and nucleotides of the plastid genomes retrieved according to datasets.

Supplementary Data 1 Annotated *de novo* reference contig (*Ouratea bahiensis*) before and after removal of the inverted repeat (in different file formats).

Supplementary Data 2 DNA sequence alignment of dataset 1.

Supplementary Data 3 DNA sequence alignment of dataset 2.

Supplementary Data 4 DNA sequence alignment of dataset 2 after trimming with trimAl.
