# Supplementary figures and images for: Resolving Recalcitrant Clades in the Pantropical Ochnaceae: Insights From Comparative Phylogenomics of Plastome and Nuclear Genomic Data Derived From Targeted Sequencing
Source: Front Plant Sci. 2021 Feb 4;12:638650. doi: 10.3389/fpls.2021.638650 (PMC7890083; doi:10.3389/fpls.2021.638650)

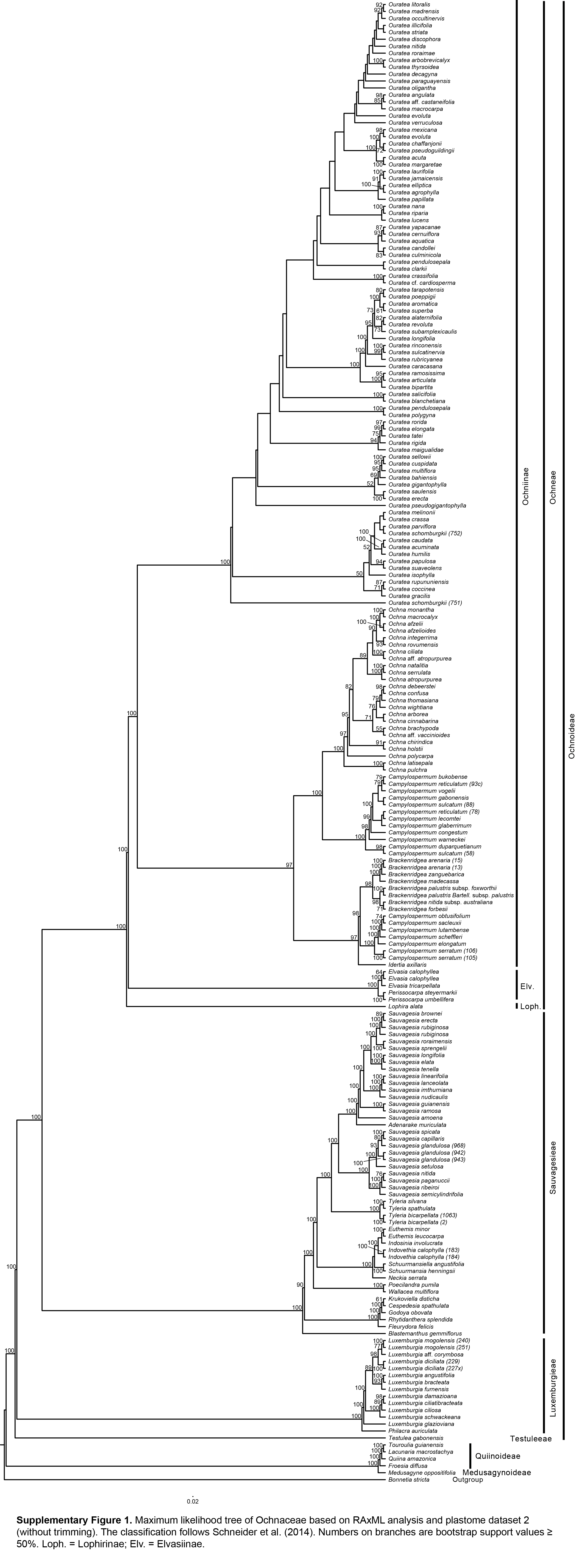

Supplement: Supplementary Figure 1 — Maximum likelihood tree of Ochnaceae based on RAxML analysis and plastome dataset 2 (without trimming). [file Image_1.JPEG]

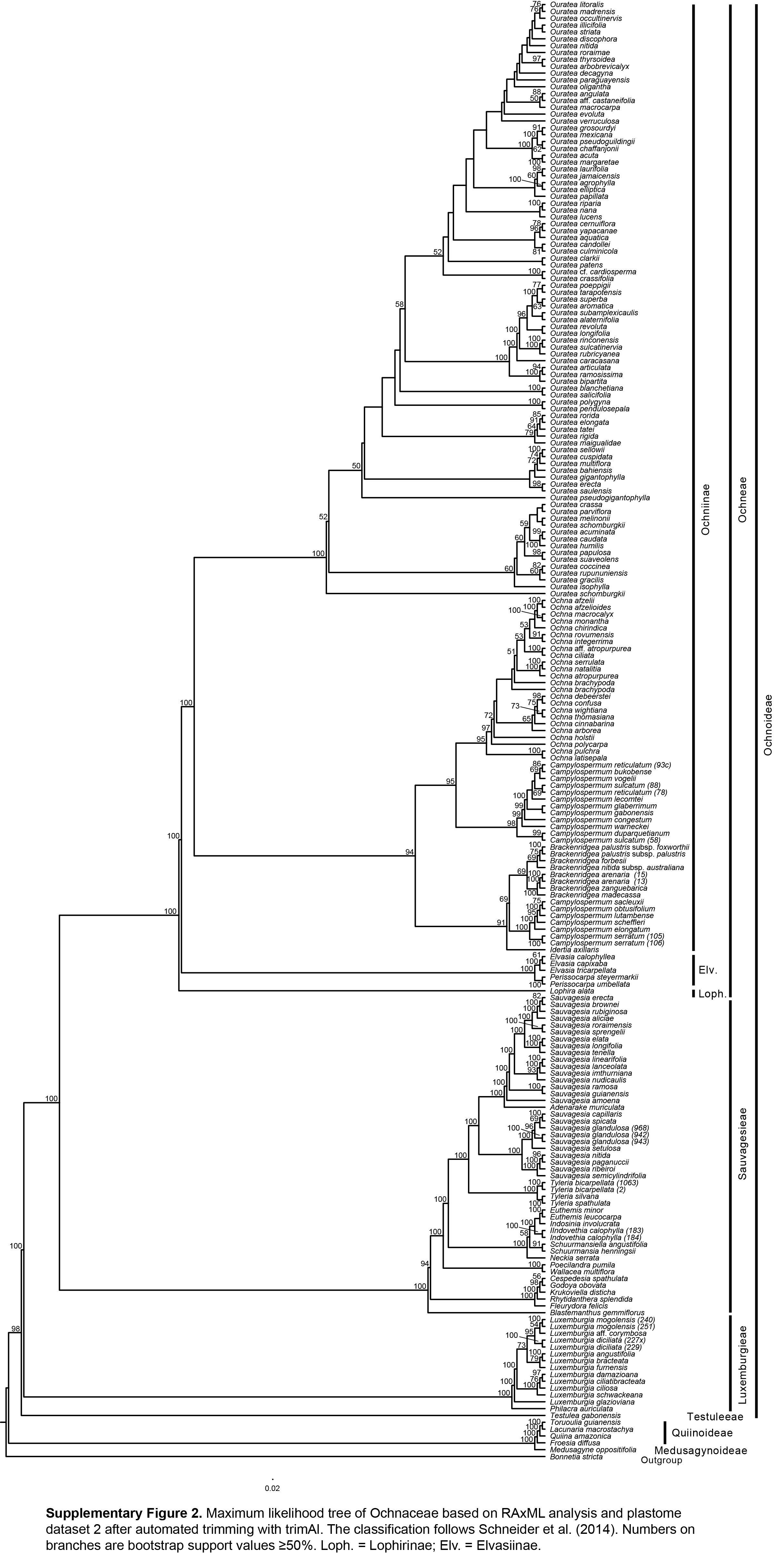

Supplement: Supplementary Figure 2 — Maximum likelihood tree of Ochnaceae based on RAxML analysis and plastome dataset 2 after automated trimming with trimAl. [file Image_2.JPEG]

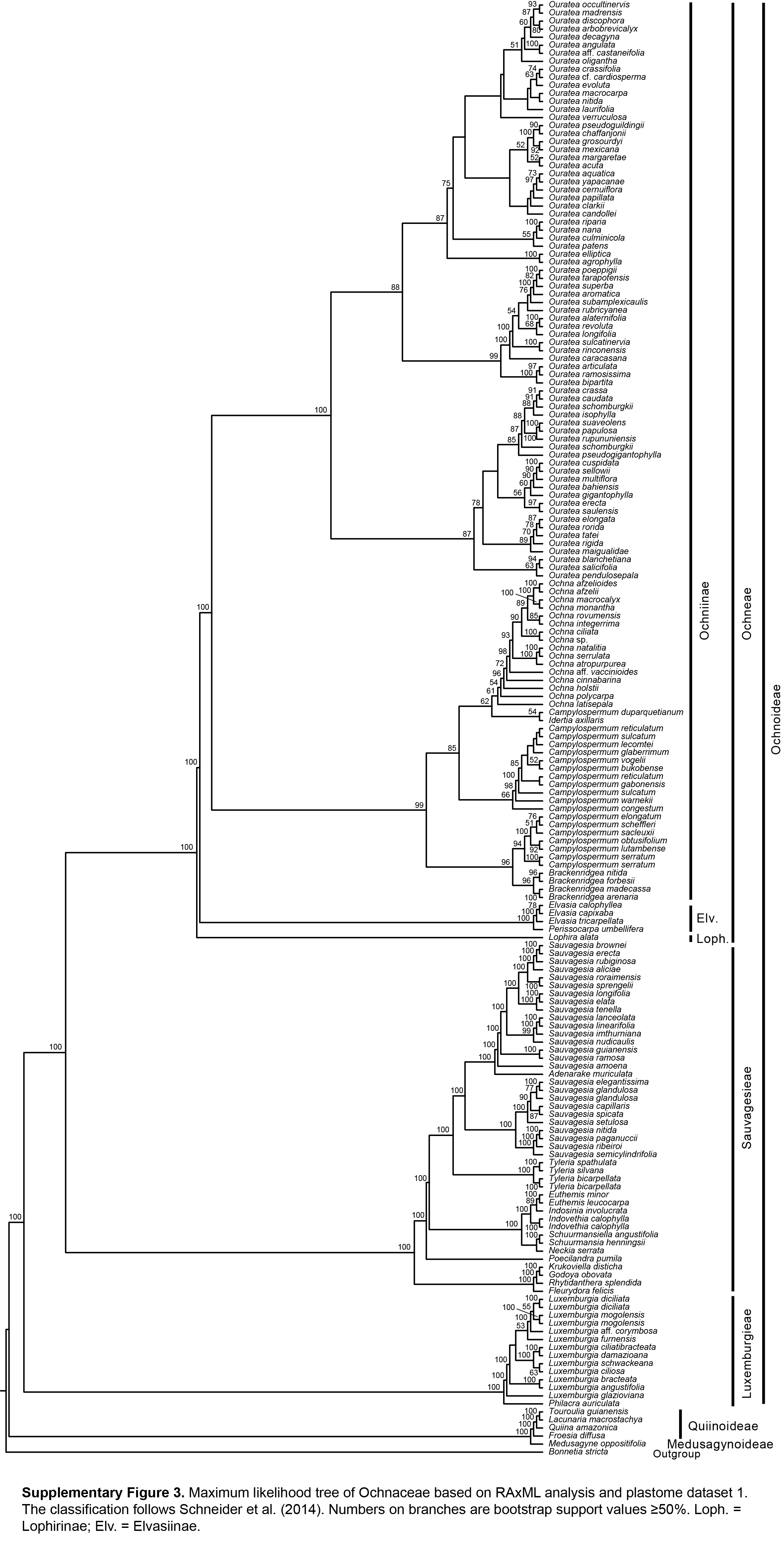

Supplement: Supplementary Figure 3 — Maximum likelihood tree of Ochnaceae based on RAxML analysis and plastome dataset 1. [file Image_3.JPEG]

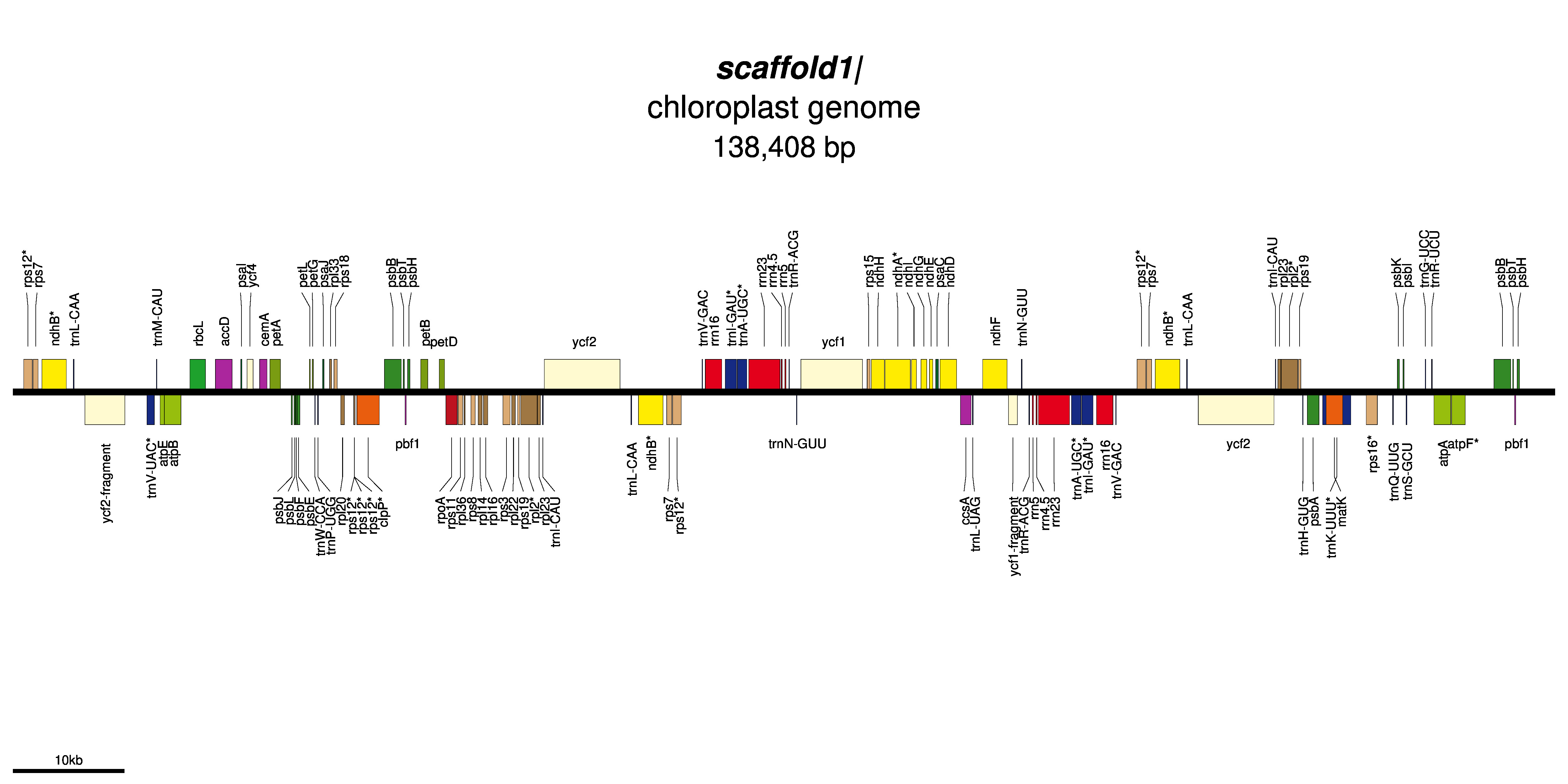

Supplement: Supplementary Data 1 — Annotated de novo reference contig (Ouratea bahiensis) before and after removal of the inverted repeat (in different file formats). [file Data_Sheet_1.ZIP › GeSeqJob-20200503-172332_scaffold1_size138408_OGDRAW.jpg]
