# Supplementary material for: Resolving Recalcitrant Clades in the Pantropical Ochnaceae: Insights From Comparative Phylogenomics of Plastome and Nuclear Genomic Data Derived From Targeted Sequencing
Source: Front Plant Sci. 2021 Feb 4;12:638650. doi: 10.3389/fpls.2021.638650 (PMC7890083; doi:10.3389/fpls.2021.638650)
Supplement: Supplementary Table 1 — List of specimens with voucher information. [file Table_1.DOCX]

| **Supplementary Table 1.** List of specimens with voucher information. | | | | |
| --- | --- | --- | --- | --- |
| **Taxon** | **Specimen (herbarium acronym)** | **Year of collection** | **Country** | **Lab ID** |
| *Adenarake muriculata* Maguire & Wurdack | Maguire, B 60447 (GH) | 1965 | Brazil | 4 |
| *Blastemanthus gemmiflorus* (Mart.) Planch. | Prance et al. 15495 (U) | 1971 | Brazil | 10 |
| *Brackenridgea arenaria* (De Wild. & T. Durand) N. Robson | Bingham, MG 8564 (WAG) | 1992 | Zambia | 13 |
| *Brackenridgea arenaria* (De Wild. & T. Durand) N. Robson | Leteinturier et al. 253 (BR) | 1998 | Zambia | 15 |
| *Brackenridgea nitida* ssp. *australiana* (F. Muell.) Kanis | Costion, C 1444 (CNS) | 2008 | Australia | 16B |
| *Brackenridgea forbesii* Tiegh. | Versteegh, C BW4816 (P) | 1958 | New Guinea | 19 |
| *Brackenridgea madecassa* (H. Perrier) Callm. | Ammann, MY 361 (P) | 2009 | Madagascar | 21 |
| *Brackenridgea palustris* Bartell. ssp. *palustris* | Podzorski, AC SMHI 547 (A) | 1984 | Philippines | 28 |
| *Brackenridgea palustris* ssp. *foxworthii* (Elm.) Kanis | Soejarto, DD et al. 6859 (WAG) | 1990 | Philippines | 20 |
| *Brackenridgea zanguebarica* Oliv. | Kathumba & Tawakali 14B (MO) | 1990 | Malawi | 34 |
| *Campylospermum bukobense* (Gilg) Farron | Gereau, R 5418 (WAG) | 1994 | DR Congo | 41 |
| *Campylospermum congestum* (Oliv.) Farron | Jongkind CCH 9158 (WAG) | 2010 | Liberia | 45 |
| *Campylospermum duparquetianum* (Baill.) Tiegh. | Dauby 2135 (MO) | 2010 | Gabon | 50 |
| *Campylospermum elongatum* (Oliv.) Tiegh. | Wieringa 6292 (WAG) | 2008 | Gabon | 55 |
| *Campylospermum gabonensis* Biss. | Bissiengou 627 (WAG) | 2009 | Gabon | 44 |
| *Campylospermum glaberrimum* (P.Beauv.) Farron | Raynal, J 13569 (P) | 1965 | Ivory coast | 303 |
| *Campylospermum lecomtei* (Tiegh.) Farron | Dechamps, R 13205 (WAG) | 1989 | Congo-Brazzaville | 82 |
| *Campylospermum lutambense* (Sleumer) Biss. | Eggeling 6416 (WAG) | 1951 | Tanzania | 46 |
| *Campylospermum obtusifolium* (Lam.) Tiegh. | Luckow, M 4101 (MO) | 1993 | Madagascar | 86 |
| *Campylospermum reticulatum* (P.Beauv.) Farron | Bissiengou 827 (WAG) | 2009 | Gabon | 78 |
| *Campylospermum reticulatum* (P.Beauv.) Farron | de Koning, J 4966 (WAG) | 1974 | Ivory coast | 93c |
| *Campylospermum sacleuxii* (Tiegh.) Farron | Mhoro, B UMBCP 409 (MO) | 2000 | Tanzania | 95 |
| *Campylospermum scheffleri* (Engl. & Gilg) Farron | Luke 7907 (MO) | 2001 | Tanzania | 96 |
| *Campylospermum serratum* (Gaertn.) Bittrich & M.C.E.Amaral | Middleton, DJ et al. 3555 (A) | 2005 | Thailand | 105 |
| *Campylospermum serratum* (Gaertn.) Bittrich & M.C.E.Amaral | T. & P. (Teo & Pachiappan) 438 (L) | 1972 | Malaysia | 106 |
| *Campylospermum sulcatum* (Tiegh.) Farron | Bissiengou 1157 (WAG) | 2010 | Gabon | 58 |
| *Campylospermum sulcatum* (Tiegh.) Farron | Bissiengou 966 (WAG) | 2010 | Gabon | 88 |
| *Campylospermum vogelii* (Hook.f. ex Planch.) Farron | Bissiengou 1058 (WAG) | 2010 | Gabon | 111 |
| *Campylospermum warneckei* (Gilg ex Engl.) Biss. | Vollesen, K 4458 (WAG) | 1977 | Tanzania | 112 |
| *Cespedesia spathulata* (Ruiz & Pav.) Planch. | Cardoso, D 3396 (HUEFS) | 2013 | Brazil | 113 |
| *Elvasia calophyllea* DC. | Kubitzki, K 84-349 (NY) | 1984 | Brazil | 126 |
| *Elvasia capixaba* Fraga & M.M. Saavedra | Zamborlini 27 (MO) | 2004 | Brazil | 131 |
| *Elvasia tricarpellata* Sastre | Santos, TS 3185 (CEPEC) | 1978 | Brazil | 146 |
| *Euthemis leucocarpa* Jack | van Niel, 4057 (L) | 1965 | Brunei | 154 |
| *Euthemis minor* Jack | Lanas et al. 789 (L) | 1997 | Indonesia | 159 |
| *Fleurydora felicis* A. Chev. | Haba, PM 18 (P) | 2007 | Guinea | 163 |
| *Froesia diffusa* Gereau & Vásquez | Zárate, R s.n. (HH) | 2013 | Peru | 167 |
| *Godoya obovata* Ruiz & Pav. | Neill & Quizphe 14978 (MO) | 2005 | Ecuador | 171 |
| *Idertia axillaris* (Oliv.) Farron | Jongkind, C.C.H. 11167 (WAG) | 2011 | Guinea | 179 |
| *Indosinia involucrata* (Gagnep.) J.E. Vidal | Poilane, E 3656 (P) | 1922 | Vietnam | 182 |
| *Indovethia calophylla* Boerl. | Brooke, W.M.A. 9671 (L) | 1955 | Malaysia | 183 |
| *Indovethia calophylla* Boerl. | van Balgooy & Setten 5315 (L) | 1986 | Indonesia | 184 |
| *Krukoviella disticha* (Tiegh.) Dwyer | Neill et al 15849 (MO) | 2007 | Ecuador | 186 |
| *Lacunaria macrostachya* (Tul.) A.C. Sm. | Zárate, R 16751 (HH) | 2011 | Peru | 193 |
| *Lophira alata* Banks ex C.F. Gaertn. | RGBE 20110701A (RBGE) | 2012 | na | 199 |
| *Luxemburgia angustifolia* Planch. | Yamamoto, K 56 (UEC) | 2002 | Brazil | 204 |
| *Luxemburgia bracteata* Dwyer | Cardoso, D 4118 (HUEFS) | 2016 | Brazil | 259 |
| *Luxemburgia ciliatibracteata* Sastre | Kinoshita, LS 00/265 (UEC) | 2000 | Brazil | 212 |
| *Luxemburgia ciliosa* (Mart.) Planch. | Feres, F 77 (UEC) | 2002 | Brazil | 217 |
| *Luxemburgia* aff. *corymbosa* A. St.-Hil. | Cardoso, D 2766 (HUEFS) | 2009 | Brazil | 252 |
| *Luxemburgia damazioana* Beauverd | Kinoshita, LS 00/225 (UEC) | 2000 | Brazil | 225 |
| *Luxemburgia* *diciliata* Dwyer | Benko-Iseppon, AM et al 1789 (UFP) | 2012 | Brazil | 227x |
| *Luxemburgia diciliata* Dwyer | Cardoso, D 3847 (HUEFS) | 2015 | Brazil | 229 |
| *Luxemburgia furnensis* Feres | Feres, F 103 (HUEFS) | 2003 | Brazil | 232 |
| *Luxemburgia glazioviana* (Engl.) Beauverd | Feres, F 99/79 (UEC) | 1999 | Brazil | 235 |
| *Luxemburgia mogolensis* Feres | Freire-Fierros, A CFCR 12462 (UEC) | 1989 | Brazil | 240 |
| *Luxemburgia* *mogolensis* Feres | Cardoso, D 2764 (HUEFS) | 2009 | Brazil | 251 |
| *Luxemburgia schwackeana* Taub. | Esteves, G.L., Morawetz & Wallnöfer s.n. (CFCR no. 15466) (LZ) | 1990 | Brazil | 248 |
| *Medusagyne oppositifolia* Baker | RGBE 20030393 (RBGE) | 2003 | Seychelles | 264 |
| *Neckia serrata* Korth. | Khairuddin (F.R.I.) 31754 (L) | 1982 | Indonesia | 267 |
| *Ochna afzelii* R. Br. ex Oliv. | Haba, OO 104 (WAG) | 2009 | Guinea | 269 |
| *Ochna afzelioides* N. Robson | Bainbridge, WR 693 (MO) | 1963 | na | 276 |
| *Ochna arborea* Burch. ex DC. | Venter 10068 (NY) | 2003 | South Africa | 280 |
| *Ochna atropurpurea* DC. | Dahlstrand, KA 1934 (GB) | 1970 | South Africa | 281 |
| *Ochna* aff. *atropurpurea* DC. | Stoffers, AL (U) | 1952 | na (cultiv.) | 285 |
| *Ochna brachypoda* Baill. | Humbert, H 24010 (P) | 1950 | Madagascar | 288 |
| *Ochna chirindica* Baker f. (= *O. holstii* Engl.) | Lanjouw 1310 (U) | 1938 | Zimbabwe | 290 |
| *Ochna ciliata* Lam. | Pascal 760 (WAG) | 1996 | Mayotte | 293 |
| *Ochna cinnabarina* Engl. & Gilg | Friederich, GR 128 (WAG) | 2001 | Namibia | 296 |
| *Ochna confusa* Burtt Davy & Greenway | Harder, DK 2156 (MO) | 1993 | Zambia | 298 |
| *Ochna debeerstei* De Wild. | Duvigneaud, P 1064 (P) | 1949 | Congo | 300 |
| *Ochna holstii* Engl. | Hafashimana, DLN 662 (K) | 1998 | Uganda | 307 |
| *Ochna integerrima* (Lour.) Merr. | Svengsuksa, B BT34 (L) | 2005 | Laos | 317 |
| *Ochna latisepala* (Tiegh.) Bamps | Niangadouma, R & Gretchen Walters 151 (MO) | 2003 | Gabon | 328 |
| *Ochna macrocalyx* Oliv. | Chandler, P 2031 (P) | 1937 | Uganda | 336 |
| *Ochna monantha* Gilg | Reekmans, M 6508 (P) | 1977 | Burundi | 345 |
| *Ochna natalitia* (Meisn.) Walp. | RBGE 19490083B (RBGE) | 2012 | BG Edinburgh | 356 |
| *Ochna polycarpa* Baker | Rakotomalaza, P.-J. et al. 1198 (WAG) | 1997 | Madagascar | 372 |
| *Ochna puberula* N. Robson | Nkhoma, CN 53 (MO) | 1993 | Zambia | 379 |
| *Ochna pulchra* Hook. | Silver C SIL29 (WAG) | 2000 | Namibia | 382 |
| *Ochna rovumensis* Gilg | Vollesen, K 4170 (WAG) | 1976 | Tanzania | 389 |
| *Ochna serrulata* (Hochst.) Walp. | BGBM XX-0-B-0500474 (BGBM) | 2016 | BG Berlin | 401 |
| *Ochna thomasiana* Engl. & Gilg | Beentje 2365 (WAG) | 1985 | Kenya | 411 |
| *Ochna aff. vaccinioides* Baker | Andriamihajarivo, T (P) | 2007 | Madagascar | 414 |
| *Ochna wightiana* Wall. | Comanor, P.L. 613 (L) | 1967 | Sri Lanka | 415 |
| *Ouratea acuminata* (DC.) Engl. | Ratter, JA et al. 437 (P) | 1967 | Brazil | 418 |
| *Ouratea acuta* (Tiegh.) Sastre | Emmerich, M 3830 (P) | 1973 | Brazil | 420 |
| *Ouratea agrophylla* (Tiegh.) Urb. | Jack, JG 6016 (P) | 1928 | Cuba | 423 |
| *Ouratea alaternifolia* (A. Rich) Engl. | Hartley, TG 13340 (P) | 1967 | Puerto Rico | 424 |
| *Ouratea angulata* Tiegh. | Hallé 4515 (U) | 1996 | French Guiana | 427 |
| *Ouratea aquatica* (Kunth) Engl. | Maguire, B & Wurdack 35574 (P) | 1953 | Venezuela | 434 |
| *Ouratea arbobrevicalyx* Sastre | Maguire, B et al. 42650 (P) | 1958 | Venezuela | 436 |
| *Ouratea aromatica* J.F. Macbr. | Vásquez, R & T. Soto 12344 (USM) | 1989 | Peru | 438 |
| *Ouratea articulata* Sastre | Sastre, C et al. 8503 (P) | 1988 | Venezuela | 439 |
| *Ouratea bahiensis* Sastre | Cardoso, D 2497 (HUEFS) | 2009 | Brazil | 842 |
| *Ouratea bipartita* Sastre | Pipoly, JJ et al. 10592 (P) | 1987 | Guyana | 445 |
| *Ouratea blanchetiana* (Planch.) Engl. | Duarte 9203 (P) | 1963 | Brazil | 446 |
| *Ouratea candollei* (Planch.) Tiegh. | Sastre, C 6448 (P) | 1978 | French Guiana | 455 |
| *Ouratea caracasana* (Planch.) Engl. | Cardozo, A et al. 2377 (P) | 1995 | Venezuela | 456 |
| *Ouratea* cf. *cardiosperma* (DC.) Engl. | Mori, SA et al. 11281 (P) | 1978 | Brazil | 457 |
| *Ouratea* aff. *castaneifolia* (DC.) Engl. | Milliken 27 (NY) | 1987 | Brazil | 464 |
| *Ouratea caudata* Engl. | Krapovickas, A 12905 (P) | 1967 | Brazil | 470 |
| *Ouratea cernuiflora* Sandwith | Pipoly, JJ & Gharbarran 9973 (MO) | 1987 | Guyana | 477 |
| *Ouratea chaffanjonii* (Tiegh.) Sastre | Sastre 8564 (U) | 1988 | Venezuela | 480c |
| *Ouratea clarkii* Sastre | Liesner, RL 7076 (P) | 1979 | Venezuela | 486 |
| *Ouratea coccinea* Engl. | Ratter, J et al. 6346 (P) | 1988 | Brazil | 488 |
| *Ouratea crassa* Tiegh. | Athayde, P (Herb Rio 109020) (P) | 1961 | Brazil | 491 |
| *Ouratea crassifolia* (Pohl) Engl. | Harley, RM & Taylor 27015 (P) | 1988 | Brazil | 492 |
| *Ouratea culminicola* Maguire & Steyerm. | Steyermark, JA et al. 117388 (P) | 1978 | Venezuela | 496 |
| *Ouratea cuspidata* (A. St.-Hil.) Engl. | Glaziou, A 11868 (P) | 1880 | Brazil | 499 |
| *Ouratea decagyna* Maguire | Prévost, MF & Sabatier 2952 (P) | 1991 | French Guiana | 502 |
| *Ouratea discophora* Ducke | Ratter, J.A. et al. 2373 (U) | 1972 | Brazil | 508 |
| *Ouratea elliptica* (A. Rich.) M. Gómez | Curtiss, AH 377 (P) | 1904 | Cuba | 497 |
| *Ouratea elongata* (Oliv.) Engl. | Sastre, C et al. 8465 (P) | 1988 | Venezuela | 518 |
| *Ouratea erecta* Sastre | Jansen-Jacobs, MJ et al 6712 (U) | 2004 | Suriname | 522 |
| *Ouratea evoluta* Maguire & Steyerm. | Richard, H 556 (P) | 2012 | French Guiana | 528c |
| *Ouratea gigantophylla* (Erhard) Engl. | Cardoso, D 2417 (HUEFS) | 2009 | Brazil | 542 |
| *Ouratea* *gracilis* D.B.O.S. Cardoso & L. Marinho | Cardoso, D 2811 (HUEFS) | 2009 | Brazil | 421 |
| *c* (Tiegh.) Steyerm. | Sastre et al. 8563 (U) | 1988 | Venezuela | 558 |
| *Ouratea humilis* Engl. | Oldenburger, F.H.F. & Mecenas 1865 (U) | 1975 | Brazil | 579 |
| *Ouratea illicifolia* (DC.) Baill. | Exk Kuba 2006 Univ Frankfurt/M 619 (FR) | 2006 | Cuba | 585 |
| *Ouratea isophylla* (Garcke) Tiegh. | Hawkins, T 1794 (P) | 1998 | Suriname | 591 |
| *Ouratea jamaicensis* (Planch.) Urb. | Acevedo-Rodriguez, P 12059 (US) | 2001 | Jamaica | 594 |
| *Ouratea laurifolia* (Sw.) Engl. | Proctor, G.R. 35608 (U) | 1976 | Jamaica | 608 |
| *Ouratea litoralis* Urb. | Wagner, R.J. 1561 (U) | 1968 | Puerto Rico | 619 |
| *Ouratea longifolia* (Lam.) Engl. | Read, RW 83-19 (US) | 1983 | Guadaloupe | 623 |
| *Ouratea lucens* (Kunth) Engl. | Huber & Weissenhofer 11.03.94-357 (LZ) | 1993 | Costa Rica | 626 |
| *Ouratea macrocarpa* Sastre | Sastre, C 5785 (P) | 1977 | French Guiana | 634 |
| *Ouratea madrensis* L. Riley | McVaugh 23002 (MO) | 1965 | Mexico | 635 |
| *Ouratea maigualidae* Sastre | Berry, PE 4876 (P) | 1991 | Venezuela | 638 |
| *Ouratea margaretae* Sastre | Sidney 198 (P) | 1967 | Brazil | 640 |
| *Ouratea melinonii* (Tiegh.) Lemée | Prévost, M.-F. 3482 (U) | 1998 | French Guiana | 644 |
| *Ouratea mexicana* (Bonpl.) Engl. | Torres & Tesorio 265 (MO) | 1982 | Mexico | 648 |
| *Ouratea multiflora* (Pohl) Engl. | Filho, AC 1465 (P) | 1983 | Brazil | 653 |
| *Ouratea nana* (A. St.-Hil.) Engl. | Irwin, HS & Soderstrom 6413 (P) | 1964 | Brazil | 654 |
| *Ouratea nitida* (Sw.) Engl. | Reveal, JL et al. 7361 (NY) | 1994 | Nicaragua | 660 |
| *Ouratea occultinervis* Sastre | Fleury, M 2160 (P) | 2006 | French Guiana | 665 |
| *Ouratea oligantha* Steyerm. ex Sastre | Pipoly, JJ & Gharbarran 10197 (P) | 1987 | Guyana | 668 |
| *Ouratea papillata* Maguire & Steyerm. | Maguire, B et al. 37663 (P) | 1954 | Venezuela | 678 |
| *Ouratea papulosa* Sastre | Mori, SA et al. 12714 (P) | 1979 | Brazil | 679 |
| *Ouratea paraguayensis* Hassl. ex Sastre & Offroy | Nee 47102 (P) | 1996 | Bolivia | 680 |
| *Ouratea parviflora* (A.DC.) Baillon | Almeida-Scabbia, RJ & Dias 5022 (P) | 2007 | Brazil | 683 |
| *Ouratea patens* Engl. | Rodrigues, W & Coelho 5625 (P) | 1963 | Brazil | 687 |
| *Ouratea pendulosepala* Sastre | Tavares, A.S. 190 (P) | 1987 | Brazil | 693 |
| *Ouratea poeppigii* Tiegh. | Mori, SA & Gracie 22365 (P) | 1992 | Brazil | 700 |
| *Ouratea polygyna* Engl. | Pires, MJP 627 (P) | 1985 | Brazil | 705 |
| *Ouratea pseudogigantophylla* Sastre | Irwin HS et al. 55082 (P) | 1963 | Suriname | 707 |
| *Ouratea pseudoguildingii* Sastre | Tillett, S et al. SPB 279 (P) | 1990 | Venezuela | 710 |
| *Ouratea ramosissima* Maguire & Steyerm. | Maguire, B & Maguire 35263 (P) | 1953 | Venezuela | 723 |
| *Ouratea revoluta* (C. Wright ex Griseb.) Engl. | Hernandez Valdes, JA & L Mecías 36977 (FR) | 1978 | Cuba | 726 |
| *Ouratea rigida* Engl. | McDowell, T et al. 1870 (P) | 1990 | Guyana | 729 |
| *Ouratea rinconensis* Whitef. | Aguilar 263 (MO) | 1991 | Costa Rica | 730 |
| *Ouratea riparia* Sleumer | Lindeman, JC et al. 240 (P) | 1980 | Suriname | 732 |
| *Ouratea roraimae* Engl. | Steyermark 113814 (P) | 1977 | Venezuela | 734 |
| *Ouratea rorida* Sastre | Redden, KM et al. 1679 (P) | 2004 | Guyana | 735 |
| *Ouratea rubricyanea* Cuatrec. | Monsalve, M 516 (P) | 1984 | Colombia | 741 |
| *Ouratea rupununiensis* Klotzsch ex Engl. | Maas et al. 7353 (U) | 1988 | Guyana | 744 |
| *Ouratea salicifolia* (A.St.-Hil. & Tul.) Engl. | Silva, RM et al. (SPF 35290) (P) | 1984 | Brazil | 746 |
| *Ouratea saulensis* Sastre | Mori, SA & Pipoly 15489 (P) | 1983 | French Guiana | 748 |
| *Ouratea schomburgkii* (Planch.) Engl. | Cid Ferreira, CA et al. 7647 (P) | 1986 | Brazil | 751 |
| *Ouratea schomburgkii* (Planch.) Engl. | Jansen-Jacobs et al. 6775 (U) | 2004 | Suriname | 752 |
| *Ouratea sellowii* (Planch.) Engl. | Hatschbach, G 687 (P) | 1947 | Brazil | 761 |
| *Ouratea semiserrata* (Mart. & Nees) Engl. | Marquete, R 4399 (P) | 2014 | Brazil | 762 |
| *Ouratea striata* (Tiegh.) Urb. | Sattler & Gutierrez C242002 (LZ) | 2002 | Cuba | 790 |
| *Ouratea suaveolens* Engl. | Cardoso, D 3511 (HUEFS) | 2015 | Brazil | 793 |
| *Ouratea subamplexicaulis* Maguire & Steyerm. | Sastre, C et al. 8545 (P) | 1988 | Venezuela | 795 |
| *Ouratea sulcatinervia* Whitef. | Mori & Kallunki 2149 (P) | 1974 | Panama | 798 |
| *Ouratea superba* Engl. | Peters, C 84-038 (P) | 1984 | Peru | 803 |
| *Ouratea tarapotensis* J.F. Macbr. | Janovec, JP et al. 2581 (P) | 2001 | Peru | 806 |
| *Ouratea tatei* Gleason | Liesner, RL 23655 (P) | 1988 | Venezuela | 807 |
| *Ouratea thyrsoidea* Engl. | Aymard, G 9286 (P) | 1991 | Venezuela | 811 |
| *Ouratea verruculosa* Engl. | Prance, GT et al. 16031 (P) | 1971 | Brazil | 820 |
| *Ouratea yapacanae* Sastre | Maguire, B et al. 41531 (P) | 1957 | Venezuela | 832 |
| *Perissocarpa steyermarkii* (Maguire) Steyerm. & Maguire | Liesner, RL & Gonzalez 10249 (MO) | 1981 | Venezuela | 857 |
| *Perissocarpa umbellifera* Steyerm. & Maguire | Amaral, MCE 1561 (NY) | 1984 | Brazil | 859 |
| *Philacra auriculata* Dwyer | Liesner, RL 16657 (MO) | 1984 | Venezuela | 862 |
| *Poecilandra pumila* Steyerm. | Hoffmann et al. 1636 (U) | 1992 | Guyana | 869 |
| *Quiina amazonica* A.C. Sm. | Zárate 16753 (HH) | 2011 | Peru | 875c |
| *Rhytidanthera splendida* (Planch.) Tiegh. | Steyermark, JA & Manara 125172 (MO) | 1981 | Venezuela | 889 |
| *Sauvagesia aliciae* Sastre | Sastre, C 2339 (U) | 1973 | Colombia | 898 |
| *Sauvagesia amoena* Ule | Campbell P22564 (MO) | 1974 | Brazil | 976 |
| *Sauvagesia brownei* Planch. ex Linden & Planch. | Lötschert 742 (FR) | 1955 | Cuba | 898c |
| *Sauvagesia capillaris* (A. St.-Hil.) Sastre | Cardoso, D 4116 (HUEFS) | 2016 | Brazil | 900 |
| *Sauvagesia elata* Benth. | Vasconcellos 10300 (L) | 1979 | Brazil | 987 |
| *Sauvagesia erecta* L. | Benko-Iseppon 1790 (UFP/FR) | 2012 | Brazil | 907x |
| *Sauvagesia glandulosa* (A. St.-Hil.) Sastre | Cardoso, D 2741 (HUEFS) | 2009 | Brazil | 968 |
| *Sauvagesia glandulosa* (A. St.-Hil.) Sastre | Cardoso, D 3942 (HUEFS) | 2016 | Brazil | 942 |
| *Sauvagesia glandulosa* (A. St.-Hil.) Sastre | Cardoso, D 3951 (HUEFS) | 2016 | Brazil | 943 |
| *Sauvagesia guianensis* (Eichler) Sastre | Liesner, RL 24969 (KPU) | 1988 | Venezuela | 972 |
| *Sauvagesia imthurniana* (Oliv.) Dwyer | Berry, P 4872 (MO) | 1991 | Venezuela | 977 |
| *Sauvagesia lanceolata* Sastre | Cardoso, D 2584 (HUEFS) | 2009 | Brazil | 929 |
| *Sauvagesia linearifolia* A. St.-Hil. | Pansarin, ER 942 (UEC) | na | Brazil | 970 |
| *Sauvagesia longifolia* Eichler | Harley, RM 56621 (HUEFS) | 2012 | Brazil | 978 |
| *Sauvagesia nitida* Zappi & E. Lucas | Conceicao, AA 1815 (HUEFS) | 2006 | Brazil | 980 |
| *Sauvagesia nudicaulis* Maguire & Wurdack | Aymard, G 9167 (MO) | 1991 | Venezuela | 979 |
| *Sauvagesia paganuccii* D.B.O.S. Cardoso & Harley | Cardoso, D 3578 (HUEFS) | 2015 | Brazil | 933 |
| *Sauvagesia ramosa* (Gleason) Sastre | Huber, O & Tillett 2878 (MO) | 1978 | Venezuela | 975 |
| *Sauvagesia ribeiroi* Harley & Giul. | Cardoso, D 3669 (HUEFS) | 2015 | Brazil | 941 |
| *Sauvagesia roraimensis* Ule | Irwin, HS 54890a (MO) | 1963 | Suriname | 973 |
| *Sauvagesia rubiginosa* A. St.-Hil. | Cardoso, D 2854 (HUEFS) | 2010 | Brazil | 969 |
| *Sauvagesia semicylindrifolia* Sastre | Cardoso, D 2658 (HUEFS) | 2015 | Brazil | 946 |
| *Sauvagesia setulosa* Queiroz-Lima & D.B.O.S. Cardoso | Cardoso, D 3920 (HUEFS) | 2016 | Brazil | 949 |
| *Sauvagesia spicata* (Glaz. ex Dwyer) Queiroz-Lima & D.B.O.S. Cardoso | Cardoso, D 2750 (HUEFS) | 2009 | Brazil | 895 |
| *Sauvagesia sprengelii* A. St.-Hil. | Jansen-Jacobs et al. 4046 (U) | 1995 | Guyana | 953 |
| *Sauvagesia tenella* Lam. | Jansen-Jacobs et al. 4702 (U) | 1995 | Guyana | 957 |
| *Schuurmansia henningsii* K. Schum. | Morawetz & Waha 13-2287 (LZ) | 1987 | Papua New Guinea | 1047 |
| *Schuurmansiella angustifolia* (Hook. f.) Hallier f. | Panero et al. 6288 (US) | 1996 | Malaysia | 1051 |
| *Testulea gabonensis* Pellegr. | Wieringa, JJ 6171 (WAG) | 2008 | Gabon | 1056 |
| *Touroulia guianensis* Aubl. | Prévost, MF 4595 (CAY) | 2002 | French Guiana | 1058 |
| *Tyleria bicarpellata* (Maguire, Steyerm. & Wurdack) M.C.E. Amaral | Huber, O et al 10214 (US) | 1985 | Venezuela | 1063 |
| *Tyleria bicarpellata* (Maguire, Steyerm. & Wurdack) M.C.E. Amaral | Steyermark J.A. et al. 128556 (U) | 1983 | Venezuela | 2 |
| *Tyleria silvana* Maguire | Liesner & Delascio 22006 (U) | 1987 | Venezuela | 1070 |
| *Tyleria spathulata* Gleason | Liesner 17731 (U) | 1985 | Venezuela | 1072 |
| *Wallacea multiflora* Ducke | Berry, PE 5926 (MO) | 1996 | Venezuela | 1082 |
| *Bonnetia stricta* (Nees) Nees & Mart. | Benko-Iseppon 1726 (UFP) | 2012 | Brazil | 1084 |
